# Association between COVID-19 booster vaccination and influenza mortality: a nationwide retrospective cohort study using the SIVEP-Gripe database in Brazil

**DOI:** 10.1007/s12026-026-09792-0

**Published:** 2026-06-22

**Authors:** Daniele Melo Sardinha, Natália Melazo Machado Neves, Tamires de Nazaré Soares, Marcos Jessé Abrahão Silva, Lidiane Assunção de Vasconcelos, Luany Rafaele da Conceição Cruz, Karina Faine Freitas Takeda, Natasha Cristina Oliveira Andrade, Suziane do Socorro dos Santos, Felipe Costa Soares, Neiva José da Luz Dias Júnior, Yan Corrêa Rodrigues, Ilma Pastana Ferreira, Mônica Custódia do Couto Abreu Pamplona, Ivonete Vieira Pereira Peixoto, Luana Nepomuceno Gondim Costa Lima

**Affiliations:** 1 Programa de Pós-Graduação em Biologia Parasitária na Amazônia, Universidade do Estado do Pará and Instituto Evandro Chagas (PPGBPA/UEPA/IEC), Belém, Pará Brazil; 2 Programa de Pós-Graduação em Epidemiologia e Vigilância em Saúde, Instituto Evandro Chagas (PPGEVS/IEC), Ananindeua, Pará Brazil; 3 Seção de Bacteriologia e Micologia, Instituto Evandro Chagas (SABMI/IEC), Ananindeua, Pará, Brazil; 4 Departamento de Saúde Integrada, Médica e professora do curso de Medicina da Universidade do Estado do Pará (UEPA), Belém, Pará, Brazil; 5 Programa de Pós-Graduação em Virologia, Instituto Evandro Chagas (PPGV/IEC), Ananindeua, Pará, Brazil; 6 Programa de Pós-Graduação em Enfermagem, Universidade Federal do Pará (PPGENF/UFPA), Belém, Pará, Brazil; 7 Programa de Pós-Graduação em Enfermagem, Universidade do Estado do Pará e Universidade Federal do Amazonas (PPGENF/UEPA/UFAM), Belém, Pará, Brazil; 8 Laboratório de Microbiologia, Unidade Acadêmica de Serra Talhada, Universidade Federal Rural de Pernambuco (UAST/UFRPE), Serra Talhada, Pernambuco, Brazil

**Keywords:** COVID-19 Vaccines, Booster Dose, Influenza, Human, Mortality, Brazil, SIVEP-Gripe, Cohort Studies, Heterologous Immunity

## Abstract

To identify clinical and epidemiological predictors of mortality among patients hospitalized with laboratory-confirmed influenza in Brazil during 2024, and to evaluate the association of vaccination and antiviral therapy with clinical outcomes. We conducted a nationwide retrospective cohort study using the Brazilian SIVEP-Gripe surveillance database. All hospitalized patients with RT-PCR–confirmed influenza and known outcomes were included. Multivariable logistic regression was initially performed to explore predictors of mortality. To more rigorously assess the association between COVID-19 booster vaccination and mortality, a propensity score–based inverse probability of treatment weighting (IPTW) approach was applied, with weights truncated at 10. E-values were calculated to assess robustness to unmeasured confounding. A total of 15,995 hospitalized influenza cases were included, with an overall case fatality rate of 12.1%. Influenza A predominated (88.0%) and was associated with higher mortality than Influenza B. In IPTW-weighted models, COVID-19 booster vaccination remained independently associated with reduced mortality (OR 0.90, 95% CI 0.84–0.97; *p* = 0.007). Additional risk factors included dyspnea, respiratory distress, and low oxygen saturation, as well as comorbidities such as obesity, diabetes, liver disease, and immunosuppression. Protective factors included influenza vaccination (OR 0.77) and oseltamivir use (OR 0.81). The model demonstrated good discrimination (AUC = 0.81, 95% CI 0.80–0.82). The E-value for the booster association was 1.46, indicating moderate robustness to unmeasured confounding. Influenza mortality in Brazil is primarily driven by age, comorbidities, and clinical severity at presentation, but is significantly mitigated by vaccination and antiviral therapy. The observed association between COVID-19 booster vaccination and reduced mortality suggests a possible heterologous immune effect, although causality cannot be established. These findings support integrated immunization strategies targeting multiple respiratory pathogens.

**Supplementary Information:**

The online version contains supplementary material available at 10.1007/s12026-026-09792-0.

## Highlights

- Influenza A predominated and was associated with higher mortality compared to Influenza B.
- Advanced age, respiratory severity markers, and chronic comorbidities were the main predictors of death.
- COVID-19 booster vaccination remained associated with reduced mortality after IPTW adjustment (OR 0.90, 95% CI 0.84–0.97).
- Sensitivity analysis (E-value = 1.46) indicates moderate robustness to unmeasured confounding.
- Influenza vaccination (OR 0.77) and oseltamivir use (OR 0.81) were independently associated with improved survival.

**Supplementary Information:**

The online version contains supplementary material available at 10.1007/s12026-026-09792-0.

## Introduction

Influenza remains an important cause of hospitalization and death worldwide, particularly among older adults and individuals with chronic comorbidities. Although vaccination and antiviral therapy are available, severe influenza continues to impose a substantial burden on health systems, especially in large middle-income countries with regional inequalities in access to care [1–3].

In Brazil, the SIVEP-Gripe surveillance system provides nationwide data on severe acute respiratory infections, allowing large-scale evaluation of laboratory-confirmed influenza cases and related outcomes. In the post-pandemic period, this surveillance system is particularly relevant because influenza, SARS-CoV-2, and other respiratory viruses continue to co-circulate, challenging clinical care and public health planning [4–6].

Emerging evidence suggests that vaccines may induce immune responses beyond their target pathogens, including non-specific or heterologous effects mediated by innate immune reprogramming. In this context, COVID-19 booster vaccination may plausibly influence the clinical course of other respiratory infections, although real-world evidence on its association with influenza mortality remains limited [7, 8].

Therefore, this study aimed to identify clinical and epidemiological predictors of mortality among hospitalized patients with RT-PCR-confirmed influenza in Brazil in 2024 and to evaluate the association between COVID-19 booster vaccination, influenza vaccination, antiviral therapy, and mortality using a nationwide real-world surveillance database.

## Methodology

### Study design

This was a retrospective cohort study utilizing national data from a single influenza epidemiological surveillance database (SIVEP-Gripe). The data are derived from the universal surveillance of Severe Acute Respiratory Syndrome (SARS). SARS is a notifiable disease in Brazil, with immediate reporting required [9]. The study followed the guidelines of the REporting of studies Conducted using Observational Routinely-collected health Data (RECORD) Statement [10].

### Case definition

Cases were defined as individuals with Influenza-like Illness (ILI) defined as fever (even if self-reported) accompanied by cough or sore throat, with symptom onset within the last 7 days—presenting with: dyspnea/respiratory distress, OR persistent chest pressure, OR O2 saturation < 95% in room air, OR bluish coloration of the lips or face. For notification purposes in SIVEP-Gripe, hospitalized SARS cases or SARS-related deaths were considered, regardless of hospitalization status [11].

### Study setting

The study was conducted in Brazil, a country of continental dimensions in South America characterized by a universal public health system (Unified Health System - SUS) and a robust epidemiological surveillance system. As the world’s fifth-largest country, Brazil possesses a vast territorial extent harboring rich geographic and hydrographic diversity, including the Amazon Basin. It is divided into five macro-regions (North, Northeast, Central-West, Southeast, and South) with distinct environmental and socioeconomic characteristics, which directly influence the nation’s economic activities and development patterns [12].

Climatically, Brazil presents a wide range of climate types, predominantly tropical. These vary from the humid equatorial climate in the Amazon (high temperatures and abundant rainfall) to the semi-arid climate in the Northeast (characterized by long droughts) and the subtropical climate in the South (with well-defined seasons and the occurrence of frost) [13, 14].

### Data sources and participants

Data were obtained from the Influenza Epidemiological Surveillance Information System (*Sistema de Informação de Vigilância Epidemiológica da Gripe* - SIVEP-Gripe), an official, open-access database managed by the Brazilian Ministry of Health that collects information on SARS cases in the country (https://opendatasus.saude.gov.br/dataset/?q=srag). Participants included individuals hospitalized for influenza in Brazil in 2024.

Inclusion criteria comprised only cases with laboratory confirmation for Influenza via RT-PCR and closed cases—i.e., those with complete information and a known outcome (death or survival), excluding ongoing cases or those with incomplete data. Exclusion criteria were cases attributed to other etiologies.

Missing data were handled using a complete-case approach, including only observations with available information for the variables included in each analysis. Records with missing outcome data were excluded from the study, as previously described. For covariates, categories labeled as “ignored” or undefined in the SIVEP-Gripe database were treated as missing and were not analyzed as separate categories to minimize misclassification bias. No imputation procedures were performed given the observational nature of the data and the large sample size.

The proportion of missing data varied substantially across variables. While demographic variables such as age and sex were complete, and COVID-19 vaccination status showed negligible missingness due to automatic integration with the National Health Data Network (RNDS), other variables particularly comorbidities and influenza vaccination status presented higher levels of missing data, reflecting manual data entry processes within the surveillance system. This pattern is consistent with known limitations of routinely collected administrative datasets. Although complete-case analysis may introduce bias if missingness is not completely at random, the consistency of the main findings and the large sample size support the robustness of the estimates [15, 16].

Regarding outcome classification, cases recorded as “death from other causes” were excluded to ensure a more specific assessment of influenza-related mortality.

### Study variables

The outcome variable (dependent) was death (binary: yes/no). The independent variables (predictors) were categorized as follows: Demographic: Age (continuous), Sex (Male/Female). Clinical/Symptoms: Cough, Fever, Dyspnea, Respiratory distress, O2 saturation < 95%, Fatigue, Sore throat, Vomiting, Diarrhea, Abdominal pain, Loss of smell, Loss of taste. Comorbidities: Chronic Cardiovascular Disease, Diabetes mellitus, Other Chronic Pneumopathy, Asthma, Chronic Neurological Disease, Immunodeficiency/Immunosuppression, Chronic Kidney Disease, Obesity, Chronic Hematologic Disease, Down Syndrome, Chronic Liver Disease. Interventions/History: Vaccinated (COVID-19 booster dose), Vaccinated (Influenza in the last campaign), Oseltamivir use, ICU admission, Invasive ventilation. Influenza Type: Influenza A, Influenza B. Subtypes/Lineages: Influenza A Subtype (A(H1N1)pdm09, A(H3N2), A not subtyped, A not subtypable, inconclusive, other); Influenza B Lineage (Victoria, Yamagata, not performed, inconclusive, other, blank).

### Statistical analysis

Descriptive statistics were calculated for the study population, stratified by clinical outcome (death vs. survivor) and influenza type (A vs. B). A detailed comparative analysis of clinical characteristics, symptoms, and outcomes across influenza types, subtypes, and lineages was performed and is presented in the Supplementary Appendix to provide additional epidemiological context. For descriptive proportions, 95% confidence intervals were calculated using the Wilson binomial method.

The Kolmogorov-Smirnov test was used to assess the normality of the age distribution. A *p*-value < 0.001 indicated that age did not follow a normal distribution, justifying the use of the median [17]. Consequently, the Mann-Whitney U test was employed to compare age medians between independent groups [18] (death vs. survivor and Influenza A vs. B), with *p*-values < 0.001 indicating statistically significant differences.

The association between COVID-19 booster vaccination and mortality was assessed using multivariable logistic regression. To control for confounding, a propensity score was estimated based on demographic characteristics, clinical variables, and comorbidities, including age, sex, signs and symptoms, and pre-existing conditions. Inverse probability of treatment weighting (IPTW) was then applied, with weights truncated at 10 to reduce the influence of extreme values, as recommended in the literature. Covariate balance after weighting was assessed using standardized mean differences to evaluate the adequacy of the propensity score adjustment. Covariate balance was considered adequate when absolute standardized mean differences were below 0.10 after weighting. To provide a transparent assessment of the propensity score adjustment, pre- and post-IPTW balance metrics were summarized in the Supplementary Appendix. Model calibration was evaluated using the Hosmer–Lemeshow goodness-of-fit test and complemented by inspection of agreement between observed and predicted mortality across risk strata [19].

Weighted models were fitted excluding variables considered potential mediators of the exposure–outcome relationship (such as intensive care unit admission and ventilatory support), in order to minimize overadjustment. Model discrimination was evaluated using the area under the receiver operating characteristic curve (AUC) based on predicted probabilities [20], and calibration was assessed using the Hosmer–Lemeshow test [21].

Multicollinearity was examined using variance inflation factors (VIFs) and condition indices [22]. Additionally, E-values were calculated for statistically significant associations as a sensitivity analysis to assess the potential impact of unmeasured confounding [23].

Analyses were performed using the Statistical Package for the Social Sciences (SPSS) software, version 26.0. A *p*-value < 0.05 was considered statistically significant in all analyses.

### Ethical aspects

This study used secondary, de-identified data from the publicly available SIVEP-Gripe database (Brazilian Ministry of Health). According to national regulations, studies using anonymized public data are exempt from institutional review board approval and informed consent requirements. All procedures were conducted in accordance with relevant ethical standards and the Declaration of Helsinki.

## Results

**Fig. 1:**
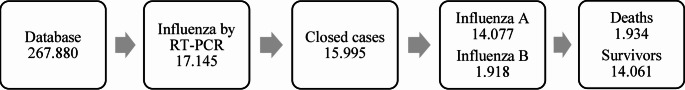
Participant selection. Source: Sivep-Gripe

Between January 1 and December 31, 2024, a total of 15,995 laboratory-confirmed influenza cases were included in the final cohort (Fig. 1). The overall case fatality rate was 12.1% (*n* = 1,934; 95% CI: 11.6–12.6%). Influenza A was the predominant type, accounting for 88.0% of cases (*n* = 14,077; 95% CI: 87.5–88.5%), and was associated with a higher case fatality rate than Influenza B: 12.7% (*n* = 1,793/14,077; 95% CI: 12.2–13.3%) versus 7.4% (*n* = 141/1,918; 95% CI: 6.3–8.6%), respectively. Among subtyped Influenza A cases, A(H1N1)pdm09 showed the highest mortality proportion, 16.8% (*n* = 702/4,177; 95% CI: 15.7–18.0%). Detailed case distributions by subtype, lineage, and mortality are presented in the Supplementary Appendix. (table S1).

The Kolmogorov–Smirnov test indicated a non-normal age distribution (*p* < 0.001). Accordingly, age was compared using the Mann–Whitney U test. Patients who died were older than survivors, with a median age of 68 years among deaths and 22 years among survivors (*p* < 0.001). Influenza A cases were also older than Influenza B cases, with median ages of 41 and 10 years, respectively (*p* < 0.001). Detailed descriptive statistics are shown in Table 1. The Mann-Whitney test (*p* < 0.001) associated older age with Influenza A, which had a mean age of 39.49 years (Median: 41; SD: 33.118; SEM: 0.279). Influenza B cases (*n* = 1,918) presented a mean age of 21.23 years (Median: 10; SD: 24.028; SEM: 0.549) (Table 1).

**Table 1:** Descriptive statistics for age (general), by outcome (death vs. survivor) and influenza type (A vs. B), in hospitalized patients in Brazil, 2024

Variables	*N* (15,995)	Mean	Median	Std. Deviation	Std. Error of Mean	Kolmogorov-Smirnov	Mann-Whitney
Age						< 0.001	
General		37.30	33.00	32.706	0.259		< 0.001
Death	1,934	62.74	68	24.186	0.550		
Survivor	14,061	33.8	22	32.173	0.271		
Influenza							< 0.001
A	14,077	39.49	41	33.118	0.279		
B	1,918	21.23	10	24.028	0.549		

Source: Sivep-Gripe

Patients with Influenza A showed a higher prevalence of respiratory severity markers than those with Influenza B, including dyspnea, low oxygen saturation, and respiratory distress. Chronic comorbidities, particularly cardiovascular disease and diabetes, were also more frequent among Influenza A cases. Conversely, fever, vomiting, and sore throat were slightly more frequent among Influenza B cases. The complete prevalence estimates, percentage differences, and graphical comparison are provided in the Supplementary Appendix. (table S2 and figure S1).

In the IPTW-weighted multivariable logistic regression model, COVID-19 booster vaccination remained independently associated with lower mortality after adjustment for demographic characteristics, clinical variables, and comorbidities (OR 0.90, 95% CI 0.84–0.97; *p* = 0.007). After weighting, covariate balance improved across most variables included in the propensity score model. Most post-weighting absolute standardized mean differences were below the conventional threshold of 0.10, indicating substantial improvement in balance between patients with and without COVID-19 booster vaccination. Residual imbalance remained for age, fever, chronic cardiovascular disease, and diabetes mellitus; therefore, these variables were retained in the final weighted outcome model to reduce residual confounding. Detailed covariate balance diagnostics are presented in Supplementary Table S3.

Older age was independently associated with higher mortality, with each additional year increasing the odds of death by approximately 2.5% (OR 1.025, 95% CI 1.024–1.027; *p* < 0.001). Male sex was also associated with increased mortality (OR 1.24, 95% CI 1.15–1.34; *p* < 0.001). Clinical markers of respiratory severity showed the strongest associations with death, including dyspnea (OR 1.70, 95% CI 1.54–1.88; *p* < 0.001), respiratory distress (OR 1.95, 95% CI 1.77–2.14; *p* < 0.001), and low oxygen saturation (OR 1.88, 95% CI 1.72–2.05; *p* < 0.001). Diarrhea was also associated with higher odds of mortality (OR 1.49, 95% CI 1.31–1.70; *p* < 0.001) (Table 2).

Among comorbidities, chronic liver disease (OR 1.51, 95% CI 1.08–2.12; *p* = 0.016), diabetes mellitus (OR 1.27, 95% CI 1.16–1.39; *p* < 0.001), chronic neurological disease (OR 1.18, 95% CI 1.02–1.36; *p* = 0.022), immunodeficiency/immunosuppression (OR 1.26, 95% CI 1.06–1.49; *p* = 0.008), and obesity (OR 1.57, 95% CI 1.35–1.83; *p* < 0.001) were independently associated with increased odds of death. These findings reinforce the role of chronic disease burden and impaired physiological reserve in influenza-related mortality (Table 2).

In contrast, some clinical manifestations were inversely associated with mortality, including fever (OR 0.83, 95% CI 0.77–0.89; *p* < 0.001), cough (OR 0.45, 95% CI 0.41–0.49; *p* < 0.001), sore throat (OR 0.85, 95% CI 0.76–0.96; *p* = 0.006), and vomiting (OR 0.81, 95% CI 0.70–0.93; *p* = 0.003). Asthma was also inversely associated with mortality (OR 0.73, 95% CI 0.63–0.85; *p* < 0.001). These inverse associations should be interpreted cautiously, as they may reflect earlier clinical presentation, differences in healthcare-seeking behavior, differential reporting, or residual confounding rather than direct protective effects (Table 2).

Preventive and therapeutic interventions were independently associated with lower mortality. Influenza vaccination in the last campaign was associated with reduced odds of death (OR 0.77, 95% CI 0.69–0.85; *p* < 0.001), as was oseltamivir use (OR 0.81, 95% CI 0.74–0.89; *p* < 0.001). COVID-19 booster vaccination showed a modest but statistically significant inverse association with mortality (OR 0.90, 95% CI 0.84–0.97; *p* = 0.007). The E-value for the booster association was 1.46, indicating moderate robustness to unmeasured confounding, while stronger E-values were observed for markers of respiratory severity, such as respiratory distress and low oxygen saturation. The corresponding odds ratios, 95% confidence intervals, p-values, and E-values are presented in Table 2.

Complementary calibration assessment showed acceptable agreement between observed and predicted mortality across quintiles of predicted risk. Predicted mortality increased progressively from the lowest to the highest risk quintile, and observed mortality followed the same gradient. Differences between observed and predicted mortality were small across strata, supporting adequate calibration of the IPTW-weighted mortality model. Detailed calibration results are presented in Supplementary Table S4.

**Fig. 2:**
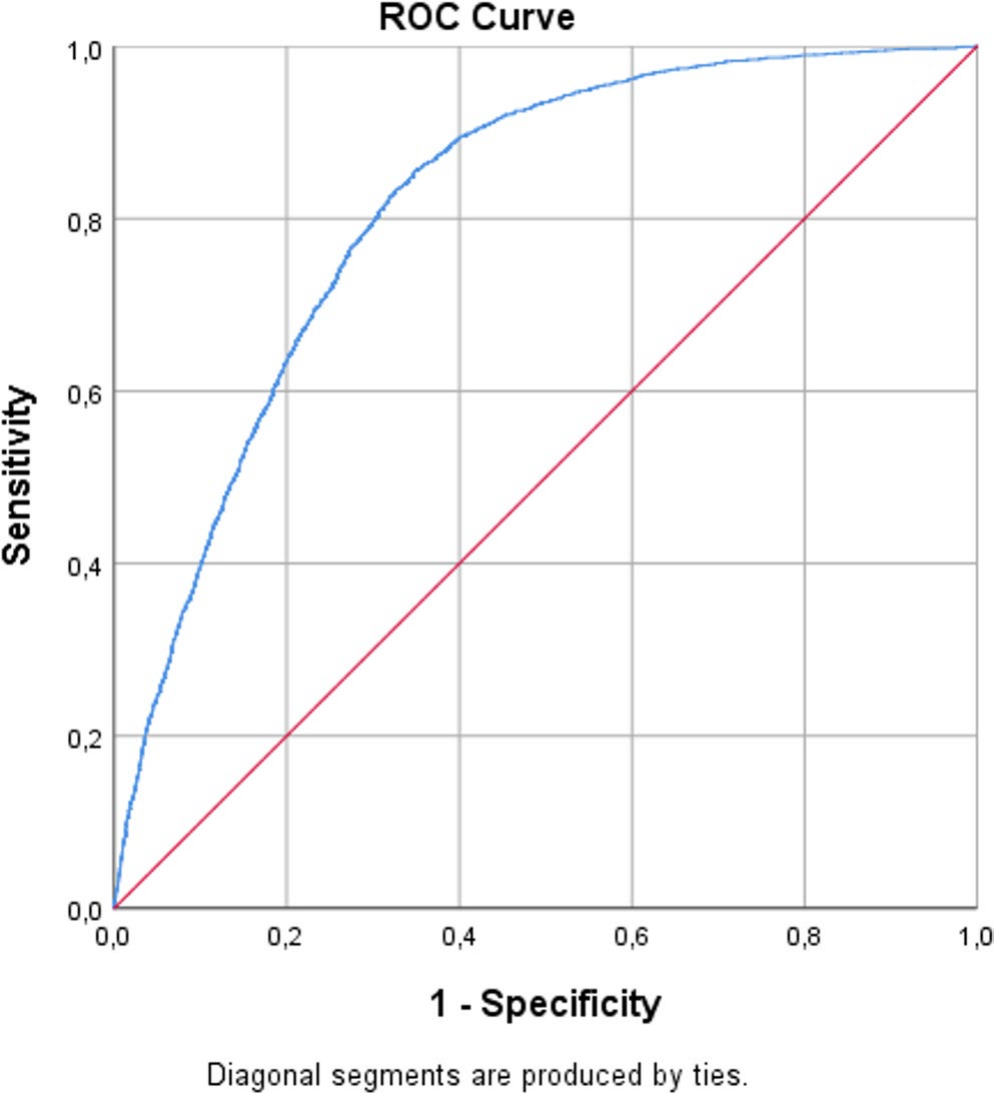
ROC curve of the final binary logistic regression model for factors associated with mortality in patients hospitalized with Influenza in Brazil, 2024. Source: Sivep-Gripe. Area under the curve (AUC) of 0.81 (95% CI 0.80–0.82; *p* < 0.001)

The model demonstrated good discrimination, with an area under the receiver operating characteristic curve of 0.81 (95% CI 0.80–0.82; *p* < 0.001), indicating adequate differentiation between individuals who died and those who survived. Although the Hosmer–Lemeshow test was statistically significant (*p* < 0.001), this result is expected in large samples and was interpreted alongside complementary calibration assessment across predicted risk strata (Fig. 2). Assessment of multicollinearity revealed no relevant concerns, with all variance inflation factors (VIFs) below 2 and condition indices below 15, confirming the stability of the estimates.

Sensitivity analyses using E-values indicated that the observed association between COVID-19 booster vaccination and mortality (E-value = 1.46) would require an unmeasured confounder with a moderate association with both the exposure and the outcome to fully explain away the effect. E-values for other significant variables ranged from approximately 1.6 to 3.3, suggesting moderate to strong robustness to unmeasured confounding. Notably, variables reflecting disease severity, such as dyspnea, respiratory distress, and low oxygen saturation, exhibited higher E-values, indicating greater resistance to residual bias.

**Table 2:** Association between clinical variables, comorbidities, and therapeutic interventions with mortality in a logistic regression model weighted by IPTW

Variable	OR	95% CI	*p*-value	E-value
Male sex	1.24	1.15–1.34	< 0.001	1.79
Age (per year)	1.025	1.024–1.027	< 0.001	1.22
Fever	0.83	0.77–0.89	< 0.001	1.70
Cough	0.45	0.41–0.49	< 0.001	3.86
Sore throat	0.85	0.76–0.96	0.006	1.61
Dyspnea	1.70	1.54–1.88	< 0.001	2.79
Respiratory distress	1.95	1.77–2.14	< 0.001	3.32
Low oxygen saturation	1.88	1.72–2.05	< 0.001	3.16
Diarrhea	1.49	1.31–1.70	< 0.001	2.34
Vomiting	0.81	0.70–0.93	0.003	1.76
Liver disease	1.51	1.08–2.12	0.016	2.39
Asthma	0.73	0.63–0.85	< 0.001	2.10
Diabetes	1.27	1.16–1.39	< 0.001	1.86
Neurological disease	1.18	1.02–1.36	0.022	1.64
Immunosuppression	1.26	1.06–1.49	0.008	1.84
Obesity	1.57	1.35–1.83	< 0.001	2.53
Influenza vaccination	0.77	0.69–0.85	< 0.001	1.92
Oseltamivir use	0.81	0.74–0.89	< 0.001	1.76
COVID-19 booster vaccination	**0.90**	**0.84–0.97**	**0.007**	**1.46**

Source: SIVEP-GRIPE. IPTW-weighted logistic regression model showing ORs, 95% CIs, and E-values for significant variables

## Discussion

This nationwide cohort study provides a comprehensive analysis of the clinical and epidemiological determinants of influenza mortality in Brazil during the 2024 season. By utilizing a continental-scale, laboratory-confirmed dataset, we provide relevant insights into the drivers of fatal outcomes in a post-pandemic context. The high proportion of missing data for comorbidities should be considered when interpreting these associations, although the main findings remained consistent across analyses.

Our findings establish that while advanced age and acute respiratory failure are the primary predictors of death, immunization specifically the inclusion of the COVID-19 booster dose alongside timely antiviral therapy with oseltamivir, significantly mitigates mortality. These results expand upon previous Brazilian surveillance data from 2020 to 2022, which identified age, absence of cough, and lack of vaccination as critical risk factors for severe acute respiratory infection (SARI) [24]. Furthermore, the observed association between age, pre-existing comorbidities (such as congestive heart failure and diabetes), and ICU admission with increased influenza A(H1N1)pdm09 mortality mirrors historical observations from the 2009 pandemic and long-term cohorts in Southern Brazil [25, 26]. Beyond these established factors, our analysis highlights a heightened vulnerability among patients with chronic liver and neurological diseases, as supported by global meta-analyses [27, 28].

The inverse associations observed for cough, fever, and asthma require cautious interpretation. Cough and fever may represent earlier or more recognizable clinical manifestations, potentially leading to faster healthcare-seeking behavior, earlier diagnostic testing, and timely initiation of supportive care or antiviral therapy. In contrast, patients presenting primarily with severe respiratory compromise, such as dyspnea, respiratory distress, or low oxygen saturation, may already be at a more advanced stage of disease at hospital admission.

The protective association observed for asthma may reflect the previously described “asthma paradox” in severe respiratory infections. Possible explanations include closer clinical monitoring, earlier access to healthcare, more frequent use of respiratory medications, and differences in inflammatory immune responses [29–31]. However, this finding should not be interpreted as evidence that asthma is intrinsically protective against influenza mortality. Residual confounding, differential reporting of comorbidities, selection bias, and incomplete clinical information in surveillance databases may also contribute to this association.

The most novel finding of this study was the independent association between COVID-19 booster vaccination and lower influenza-related mortality after IPTW adjustment (OR 0.90, 95% CI 0.84–0.97). Although the magnitude of this association was modest, its persistence after propensity score weighting and exclusion of potential mediators strengthens the robustness of the finding. The E-value of 1.46 indicates moderate resistance to unmeasured confounding, suggesting that an unmeasured factor would need to be associated with both booster vaccination and mortality by at least this magnitude to fully explain away the observed association. While previous studies have mainly evaluated whether influenza vaccination may reduce COVID-19 severity through trained immunity mechanisms [32–35], our findings raise the complementary hypothesis that COVID-19 booster vaccination may be associated with improved outcomes in influenza. This hypothesis is biologically plausible, as heterologous vaccine effects may occur through functional reprogramming of innate immune cells mediated by epigenetic and metabolic modifications [36, 37]. Nevertheless, because this was an observational study, causality cannot be established. The association may also reflect differences in healthcare access, preventive behavior, vaccination adherence, or residual confounding not fully captured in the surveillance database.

Influenza remains a persistent global health challenge, particularly as populations age and the burden of cardiopulmonary comorbidities increases [38–40]. In middle-income nations like Brazil, this burden is exacerbated by disparities in healthcare access and infrastructure [41]. Our findings underscore that strengthening influenza surveillance and maintaining high vaccine coverage are critical components of a resilient health infrastructure. The identified survival benefit of oseltamivir (OR 0.81) further reinforces the necessity of ensuring decentralized and rapid access to antiviral therapy within public health systems.

This study has several limitations that should be considered when interpreting the findings. First, as an observational study based on routinely collected surveillance data, residual confounding cannot be fully excluded. Individuals who received COVID-19 booster vaccination may differ systematically from those who did not, particularly regarding healthcare-seeking behavior, access to medical services, and adherence to preventive and therapeutic interventions.

To reduce bias, we applied propensity score–based inverse probability of treatment weighting (IPTW) and conducted sensitivity analyses using E-values. However, unmeasured or inadequately captured variables such as timing of vaccination, timing of antiviral therapy, and healthcare system factors may still influence the observed associations.

To minimize overadjustment, downstream markers of disease severity, such as intensive care unit admission and invasive mechanical ventilation, were excluded from the weighted models. While this approach preserves the interpretability of exposure effects, it may also limit the ability to fully account for the clinical trajectory of severe cases.

Additionally, the use of secondary surveillance data may introduce misclassification or incomplete reporting of clinical variables and comorbidities. Although we restricted the analysis to RT-PCR–confirmed cases to ensure diagnostic specificity, underreporting and missing data are inherent limitations of large administrative datasets. However, the proportion of excluded cases due to missing outcomes (6.7%) remained below commonly accepted thresholds for substantial bias [42].

Finally, although the observed association between COVID-19 booster vaccination and reduced mortality is biologically plausible and supported by sensitivity analyses, causal inference cannot be established, and the findings should be interpreted as associations rather than direct effects.

## Conclusion

Influenza mortality among hospitalized patients in Brazil during 2024 was mainly associated with advanced age, chronic comorbidities, and respiratory severity at presentation, particularly dyspnea, respiratory distress, and low oxygen saturation. These findings reinforce the importance of early recognition of high-risk patients at hospital admission and timely implementation of clinical management strategies.

Beyond established predictors of mortality, this nationwide cohort identified independent protective associations for influenza vaccination, oseltamivir use, and COVID-19 booster vaccination after IPTW adjustment. Although the association between COVID-19 booster vaccination and lower influenza-related mortality was modest and cannot be interpreted as causal, it raises an important hypothesis regarding potential heterologous immune benefits in the post-pandemic period.

From a public health perspective, our findings support integrated respiratory virus prevention strategies, combining high vaccination coverage, strengthened epidemiological surveillance, and equitable access to antiviral therapy. Future prospective studies and causal inference analyses are needed to clarify whether COVID-19 booster vaccination contributes to reduced influenza severity through heterologous immune mechanisms or reflects broader patterns of healthcare access and preventive behavior.

## Supplementary Information

Below is the link to the electronic supplementary material.

Supplementary Material 1

Supplementary Material 2

## Data Availability

The data are publicly and universally available on the OpenDataSUS platform (https://opendatasus.saude.gov.br/dataset/?q=srag).
