# Supplementary material for: Association between COVID-19 booster vaccination and influenza mortality: a nationwide retrospective cohort study using the SIVEP-Gripe database in Brazil
Source: Immunol Res. 2026 Jun 22;74(1):62. doi: 10.1007/s12026-026-09792-0 (PMC13284014; doi:10.1007/s12026-026-09792-0)
Supplement: Supplementary file 1 — Supplementary Material 1 [file 12026_2026_9792_MOESM1_ESM.docx]

**The RECORD statement – checklist of items, extended from the STROBE statement, that should be reported in observational studies using routinely collected health data.**

|  | **Item No.** | **STROBE items** | **Location in manuscript where items are reported** | **RECORD items** | **Location in manuscript where items are reported** |
| --- | --- | --- | --- | --- | --- |
| **Title and abstract** | | | | | |
|  | 1 | (a) Indicate the study’s design with a commonly used term in the title or the abstract (b) Provide in the abstract an informative and balanced summary of what was done and what was found |  | RECORD 1.1: The type of data used should be specified in the title or abstract. When possible, the name of the databases used should be included.  RECORD 1.2: If applicable, the geographic region and timeframe within which the study took place should be reported in the title or abstract.  RECORD 1.3: If linkage between databases was conducted for the study, this should be clearly stated in the title or abstract. | Specified in the title ("real-world cohort study - SIVEP-GRIPE") and in the abstract ("SIVEP-Gripe database").  Specified in the title ("Brazil during 2024") and in the abstract ("Brazil during the 2024 season").  Not applicable. The study utilized a single, comprehensive database (SIVEP-Gripe) without linkage to other secondary sources. |
| **Introduction** | | | | | |
| Background rationale | 2 | Explain the scientific background and rationale for the investigation being reported |  |  | Influenza causes a significant global burden of respiratory disease, with the A(H1N1)pdm09 virus remaining a persistent concern. In Brazil, despite the universal surveillance system (SIVEP-Gripe), vaccine coverage for high-risk groups remains suboptimal (50.68% as of late 2025). There is a critical need to understand mortality predictors in a post-pandemic context using large-scale, real-world data to guide clinical management and public health policies. |
| Objectives | 3 | State specific objectives, including any prespecified hypotheses |  |  | he objective was to identify demographic, clinical, and epidemiological factors associated with mortality in patients hospitalized with influenza in Brazil in 2024. The implicit hypothesis was that while advanced age and chronic comorbidities increase mortality risk, vaccination (both influenza and COVID-19) and antiviral therapy significantly mitigate this outcome |
| **Methods** | | | | | |
| Study Design | 4 | Present key elements of study design early in the paper |  |  | Retrospective cohort study using routinely collected national epidemiological surveillance data (universal surveillance of Severe Acute Respiratory Syndrome - SARS). |
| Setting | 5 | Describe the setting, locations, and relevant dates, including periods of recruitment, exposure, follow-up, and data collection |  |  | Brazil (continental dimensions, five macro-regions). The timeframe for data collection and follow-up covered the entire year of 2024 (January 1st to December 31st). |
| Participants | 6 | *(a) Cohort study* - Give the eligibility criteria, and the sources and methods of selection of participants. Describe methods of follow-up  *Case-control study* - Give the eligibility criteria, and the sources and methods of case ascertainment and control selection. Give the rationale for the choice of cases and controls  *Cross-sectional study* - Give the eligibility criteria, and the sources and methods of selection of participants  *(b) Cohort study* - For matched studies, give matching criteria and number of exposed and unexposed  *Case-control study* - For matched studies, give matching criteria and the number of controls per case |  | RECORD 6.1: The methods of study population selection (such as codes or algorithms used to identify subjects) should be listed in detail. If this is not possible, an explanation should be provided.  RECORD 6.2: Any validation studies of the codes or algorithms used to select the population should be referenced. If validation was conducted for this study and not published elsewhere, detailed methods and results should be provided.  RECORD 6.3: If the study involved linkage of databases, consider use of a flow diagram or other graphical display to demonstrate the data linkage process, including the number of individuals with linked data at each stage. | Selection was based on: 1) Notification in SIVEP-Gripe; 2) Hospitalization for SARS; 3) Laboratory confirmation via RT-PCR for Influenza; 4) "Closed cases" with a definitive clinical outcome (death or recovery).  The study utilized the official Ministry of Health system. Diagnostic validation was ensured by the inclusion criterion restricted to RT-PCR results (the gold standard).  Not applicable (single database). |
| Variables | 7 | Clearly define all outcomes, exposures, predictors, potential confounders, and effect modifiers. Give diagnostic criteria, if applicable. |  | RECORD 7.1: A complete list of codes and algorithms used to classify exposures, outcomes, confounders, and effect modifiers should be provided. If these cannot be reported, an explanation should be provided. | Standardized variables from the national SARS notification form were used: age, sex, symptoms (fever, cough, dyspnea, etc.), comorbidities (cardiovascular, diabetes, liver disease, etc.), vaccination status, and Oseltamivir use. The outcome was classified by the "Case Evolution" field (death or discharge). |
| Data sources/ measurement | 8 | For each variable of interest, give sources of data and details of methods of assessment (measurement).  Describe comparability of assessment methods if there is more than one group |  |  | Sources: Compulsory notifications entered by healthcare professionals nationwide. Measurement: Clinical data derived from medical records or self-reports, and official laboratory RT-PCR results.  All groups (Influenza A and B) were notified under the same national SARS surveillance protocol. |
| Bias | 9 | Describe any efforts to address potential sources of bias |  |  | Selection bias was mitigated by using only RT-PCR confirmed cases. Confounding was addressed via multivariate logistic regression and sensitivity analysis using the E-value. |
| Study size | 10 | Explain how the study size was arrived at |  |  | The sample size (n=15,995) was determined by the total number of cases meeting inclusion criteria in the national database during 2024 (a census approach for the period). |
| Quantitative variables | 11 | Explain how quantitative variables were handled in the analyses. If applicable, describe which groupings were chosen, and why |  |  | The main quantitative variable was age.  Treatment: Normality was tested using the Kolmogorov-Smirnov test (p < 0.001), which indicated a non-normal distribution. Therefore, the median and Mann-Whitney U test were used for comparison between groups.  Groupings: In logistic regression, age was treated as a continuous variable (OR 1.034), indicating an increased risk for each additional year of life. In the descriptive tables, data were stratified by outcome (death vs. survivor) and virus type (A vs. B). |
| Statistical methods | 12 | (a) Describe all statistical methods, including those used to control for confounding  (b) Describe any methods used to examine subgroups and interactions  (c) Explain how missing data were addressed  (d) *Cohort study* - If applicable, explain how loss to follow-up was addressed  *Case-control study* - If applicable, explain how matching of cases and controls was addressed  *Cross-sectional study* - If applicable, describe analytical methods taking account of sampling strategy  (e) Describe any sensitivity analyses |  |  | a) Multivariate binary logistic regression with stepwise backward elimination.  (b) Descriptive analysis stratified by Influenza type (A vs B) and subtypes (H1N1, H3N2, Victoria, etc.).  (c) Missing Data: 6.7% (n=1,150) of confirmed cases lacked outcome data and were excluded (complete case analysis). This was justified by the low percentage (<10% threshold).  (d) Retrospective cohort: Loss to follow-up was minimized by including only "closed cases" with a known outcome in the system.  (e) Sensitivity analyses included E-value calculation for vaccination effects and ROC Curve (AUC) to assess the model's discriminatory power. |
| Data access and cleaning methods |  | .. |  | RECORD 12.1: Authors should describe the extent to which the investigators had access to the database population used to create the study population.  RECORD 12.2: Authors should provide information on the data cleaning methods used in the study. | Authors had full access to the public, anonymized database through the OpenDataSUS platform.  Data cleaning involved filtering for RT-PCR confirmed influenza cases and removing duplicates or "open cases" (those without a final discharge or death record). |
| Linkage |  | .. |  | RECORD 12.3: State whether the study included person-level, institutional-level, or other data linkage across two or more databases. The methods of linkage and methods of linkage quality evaluation should be provided. | Not applicable. |
| **Results** | | | | | |
| Participants | 13 | (a) Report the numbers of individuals at each stage of the study (*e.g.*, numbers potentially eligible, examined for eligibility, confirmed eligible, included in the study, completing follow-up, and analysed)  (b) Give reasons for non-participation at each stage.  (c) Consider use of a flow diagram |  | RECORD 13.1: Describe in detail the selection of the persons included in the study (*i.e.,* study population selection) including filtering based on data quality, data availability and linkage. The selection of included persons can be described in the text and/or by means of the study flow diagram. | Detailed in Figure 1: 267,880 total records -> 17,145 RT-PCR confirmed -> 15,995 analyzed after excluding cases without clinical outcomes. |
| Descriptive data | 14 | (a) Give characteristics of study participants (*e.g.*, demographic, clinical, social) and information on exposures and potential confounders  (b) Indicate the number of participants with missing data for each variable of interest  (c) *Cohort study* - summarise follow-up time (*e.g.*, average and total amount) |  |  | (a) Characteristics: The profile included demographic variables (age, sex), clinical variables (symptoms such as dyspnea, fever), comorbidities (diabetes, cardiovascular diseases, etc.) and interventions (ICU, ventilation, oseltamivir).  (b) Missing Data: Reported mainly in the Influenza B lineage typing.  (c) Outcomes: 1,934 deaths and 14,061 survivors were reported. The case fatality rate was higher in Influenza A(H1N1)pdm09 (16.81%). |
| Outcome data | 15 | *Cohort study* - Report numbers of outcome events or summary measures over time  *Case-control study* - Report numbers in each exposure category, or summary measures of exposure  *Cross-sectional study* - Report numbers of outcome events or summary measures |  |  | Outcomes: 1,934 deaths and 14,061 survivors were reported. The case fatality rate was higher in Influenza A(H1N1)pdm09 (16.81%). |
| Main results | 16 | (a) Give unadjusted estimates and, if applicable, confounder-adjusted estimates and their precision (e.g., 95% confidence interval). Make clear which confounders were adjusted for and why they were included  (b) Report category boundaries when continuous variables were categorized  (c) If relevant, consider translating estimates of relative risk into absolute risk for a meaningful time period |  |  | Estimates and Precision: Confounder-adjusted estimates are provided in Table 4 as Adjusted Odds Ratios (aOR) with their respective 95% Confidence Intervals (95% CI). For example, invasive ventilation showed an aOR of 10.635 (95% CI: 9.178–12.323), while the COVID-19 booster dose showed an aOR of 0.867 (95% CI: 0.762–0.986).  Confounders Adjusted: The final multivariate model adjusted for age, sex, clinical symptoms (cough, fever, dyspnea, respiratory distress, O2 saturation, diarrhea), comorbidities (liver disease, neurological disease, immunodeficiency, asthma), and interventions/history (ICU admission, invasive ventilation, oseltamivir use, influenza vaccination, and COVID-19 booster).  Why Included: These variables were included because they are biologically and clinically recognized in the literature as factors that influence either the likelihood of vaccination (exposure) or the risk of respiratory failure and death (outcome), thereby acting as potential confounders. The selection was further refined using stepwise backward elimination to ensure the most parsimonious and statistically significant model.  Age: In this study, the continuous variable "Age" was not categorized. It was analyzed as a continuous predictor in the logistic regression model to maintain statistical power and avoid arbitrary thresholds. The resulting a OR (1.034) represents the increase in the odds of death for each additional year of life.  Other Variables: All other predictors (symptoms, comorbidities, and vaccines) were inherently categorical (binary: yes/no), so no boundaries were required.  Absolute Risk (Case Fatality Rate): The relative estimates (ORs) are complemented by absolute risk measures presented as Case Fatality Rates (CFR).  Meaningful Findings:  The overall absolute risk of death for hospitalized influenza patients in Brazil in 2024 was 12.1% (1,934/15,995).  When stratified by virus type, the absolute risk was higher for Influenza A (12.74%) compared to Influenza B (7.35%).  The highest absolute risk among subtyped cases was observed for A(H1N1)pdm09, with a fatality rate of 16.81%.  These absolute values provide a clear clinical context for the burden of disease within the hospital setting during the 2024 season. |
| Other analyses | 17 | Report other analyses done—e.g., analyses of subgroups and interactions, and sensitivity analyses |  |  | Prevalence comparison between Influenza A and B (Table 3/Figure 2) and E-value sensitivity analysis (1.57). |
| **Discussion** | | | | | |
| Key results | 18 | Summarise key results with reference to study objectives |  |  | Advanced age, invasive ventilation (OR 10.6), ICU admission, and liver/neurological diseases were the primary risk predictors. Vaccination (Influenza and COVID-19 Booster) and Oseltamivir were confirmed as significant protective factors. |
| Limitations | 19 | Discuss limitations of the study, taking into account sources of potential bias or imprecision. Discuss both direction and magnitude of any potential bias |  | RECORD 19.1: Discuss the implications of using data that were not created or collected to answer the specific research question(s). Include discussion of misclassification bias, unmeasured confounding, missing data, and changing eligibility over time, as they pertain to the study being reported. | The discussion acknowledges that while surveillance data (Real-World Data) may be subject to underreporting or clerical errors, the massive sample size (n=15,995) and the use of the RT-PCR gold standard provide robust evidence for clinical risk assessment. |
| Interpretation | 20 | Give a cautious overall interpretation of results considering objectives, limitations, multiplicity of analyses, results from similar studies, and other relevant evidence |  |  | Mortality is multifactorial. A novel and pivotal finding is the protective effect of the COVID-19 booster dose (OR 0.867), likely mediated by trained immunity (non-specific innate immune memory). |
| Generalisability | 21 | Discuss the generalisability (external validity) of the study results |  |  | The results are highly generalizable to the population hospitalized with influenza in Brazil and provide a "mirror" for upcoming seasons in other regions. |
| **Other Information** | | | | | |
| Funding | 22 | Give the source of funding and the role of the funders for the present study and, if applicable, for the original study on which the present article is based |  |  | The study received no funding. |
| Accessibility of protocol, raw data, and programming code |  | .. |  | RECORD 22.1: Authors should provide information on how to access any supplemental information such as the study protocol, raw data, or programming code. | Raw data is universally accessible via the OpenDataSUS platform (link provided in the manuscript). Analysis followed RECORD guidelines and Brazilian data protection laws (LGPD). |

*Reference: Benchimol EI, Smeeth L, Guttmann A, Harron K, Moher D, Petersen I, Sørensen HT, von Elm E, Langan SM, the RECORD Working Committee. The REporting of studies Conducted using Observational Routinely-collected health Data (RECORD) Statement. *PLoS Medicine* 2015; in press.

*Checklist is protected under Creative Commons Attribution ([CC BY](http://creativecommons.org/licenses/by/4.0/)) license.
