# Supplementary material for: Association between COVID-19 booster vaccination and influenza mortality: a nationwide retrospective cohort study using the SIVEP-Gripe database in Brazil
Source: Immunol Res. 2026 Jun 22;74(1):62. doi: 10.1007/s12026-026-09792-0 (PMC13284014; doi:10.1007/s12026-026-09792-0)
Supplement: Supplementary file 2 — Supplementary Material 2 [file 12026_2026_9792_MOESM2_ESM.docx]

**Supplementary Appendix**

Table S1 presents the distribution of Influenza A subtypes and Influenza B lineages among hospitalized patients with RT-PCR-confirmed influenza in Brazil in 2024, stratified by clinical outcome. Among Influenza A cases, “not subtyped” represented the largest group, accounting for 33.46% of cases, followed by A(H1N1)pdm09 and A(H3N2), which accounted for 29.67% and 29.03%, respectively. Together, A(H1N1)pdm09 and A(H3N2) represented nearly 60% of Influenza A cases with specific subtype classification.

A(H1N1)pdm09 showed the highest mortality proportion among Influenza A subtypes, with 702 deaths among 4,177 cases, corresponding to a mortality rate of 16.81% (95% CI: 15.70–17.97). The mortality proportion for A(H3N2) was 12.14% (95% CI: 11.17–13.18), whereas Influenza A “not subtyped” and “not subtypable” presented lower mortality estimates of 10.17% (95% CI: 9.34–11.07) and 8.90% (95% CI: 6.68–11.78), respectively. Inconclusive Influenza A cases had a mortality proportion of 8.82% (95% CI: 6.00–12.79), while the “other” category showed a mortality proportion of 14.04% (95% CI: 10.79–18.08) (table S1).

Among Influenza B cases, lineage testing was most frequently classified as “not performed”, representing 56.05% of cases, followed by missing lineage data, which accounted for 32.69%. The Victoria lineage was the most frequently identified specific Influenza B lineage, representing 7.40% of cases. Mortality estimates among Influenza B categories were generally lower than those observed for Influenza A subtypes. The Victoria lineage had a mortality proportion of 7.75% (95% CI: 4.38–13.34), and the “not performed” category had a similar mortality proportion of 7.53% (95% CI: 6.10–9.27). Cases with missing lineage data presented a mortality proportion of 7.34% (95% CI: 5.55–9.65) (table S1).

The Yamagata lineage was identified in only one case and had no deaths, resulting in a mortality estimate of 0.00% with a wide 95% confidence interval (0.00–79.35), reflecting the extreme imprecision associated with the very small sample size. Similarly, the “other” Influenza B category showed a mortality proportion of 10.00% (95% CI: 2.79–30.10), but this estimate should be interpreted cautiously due to the limited number of cases. Overall, the confidence intervals reinforce that mortality estimates were more precise in larger subtype or lineage groups and substantially less precise in categories with small denominators (table S1).

**Table S1** – Distribution of Influenza A subtypes and Influenza B lineages among hospitalized patients with RT-PCR-confirmed influenza in Brazil, 2024, according to clinical outcome.

| Influenza subtype/lineage | Survivors n (%) | Deaths n (%) | Total n (%) | Mortality 95% CI |
| --- | --- | --- | --- | --- |
| Influenza A subtype |  |  |  |  |
| Influenza A(H1N1)pdm09 | 3,475 (83.19) | 702 (16.81) | 4,177 (29.67) | 15.70–17.97 |
| Influenza A(H3N2) | 3,590 (87.86) | 496 (12.14) | 4,086 (29.03) | 11.17–13.18 |
| Influenza A not subtyped | 4,231 (89.83) | 479 (10.17) | 4,710 (33.46) | 9.34–11.07 |
| Influenza A not subtypable | 440 (91.10) | 43 (8.90) | 483 (3.43) | 6.68–11.78 |
| Inconclusive | 248 (91.18) | 24 (8.82) | 272 (1.93) | 6.00–12.79 |
| Other | 300 (85.96) | 49 (14.04) | 349 (2.48) | 10.79–18.08 |
| Influenza B lineage |  |  |  |  |
| Victoria | 131 (92.25) | 11 (7.75) | 142 (7.40) | 4.38–13.34 |
| Yamagata | 1 (100.00) | 0 (0.00) | 1 (0.05) | 0.00–79.35 |
| Not performed | 994 (92.47) | 81 (7.53) | 1,075 (56.05) | 6.10–9.27 |
| Inconclusive | 52 (98.11) | 1 (1.89) | 53 (2.76) | 0.33–9.94 |
| Other | 18 (90.00) | 2 (10.00) | 20 (1.04) | 2.79–30.10 |
| Missing data | 581 (92.66) | 46 (7.34) | 627 (32.69) | 5.55–9.65 |

**Legend:** Percentages for survivors and deaths were calculated within each subtype or lineage category. Total percentages were calculated within Influenza A or Influenza B groups, respectively. Mortality 95% confidence intervals were calculated using the Wilson binomial method. Influenza A “not subtyped” refers to RT-PCR-confirmed Influenza A cases for which subtype classification, such as A(H1N1)pdm09 or A(H3N2), was not reported. “Not subtypable” refers to cases in which subtyping was attempted but could not be technically assigned. For Influenza B, “not performed” indicates that lineage testing was not conducted, whereas “missing data” indicates absence of recorded lineage information in the database. Source: SIVEP-Gripe.

Table S2 and Figure S1 presents the prevalence of demographic characteristics, symptoms, respiratory severity markers, comorbidities, preventive and therapeutic interventions, and clinical outcomes among hospitalized patients with Influenza A and Influenza B in Brazil in 2024. The table was organized into thematic blocks to improve readability and facilitate interpretation of differences between influenza types.

In the demographic block, the proportion of male patients was similar between Influenza A and Influenza B cases, with prevalences of 48.48% (95% CI: 47.66–49.31) and 48.70% (95% CI: 46.46–50.93), respectively. This small difference suggests that sex distribution was comparable between both influenza types (table S2).

In the symptoms and respiratory severity block, Influenza A cases showed higher prevalence of markers associated with respiratory compromise. Dyspnea was reported in 62.68% of Influenza A cases (95% CI: 61.88–63.48), compared with 49.37% of Influenza B cases (95% CI: 47.14–51.61), corresponding to a difference of 13.31 percentage points. Low oxygen saturation was also more frequent among Influenza A cases, with a prevalence of 52.55% (95% CI: 51.73–53.38), compared with 42.18% (95% CI: 39.99–44.40) among Influenza B cases. Respiratory distress followed the same pattern, occurring in 59.96% of Influenza A cases (95% CI: 59.15–60.77) and 53.13% of Influenza B cases (95% CI: 50.89–55.35) (table S2).

Conversely, some symptoms were more frequent among Influenza B cases. Fever was observed in 76.90% of Influenza B cases (95% CI: 74.96–78.73), compared with 71.66% of Influenza A cases (95% CI: 70.91–72.39). Vomiting and sore throat were also more prevalent in Influenza B, with differences of -3.86 and -2.57 percentage points, respectively. These findings suggest that Influenza B cases presented proportionally more constitutional or upper respiratory/gastrointestinal symptoms, whereas Influenza A cases showed a greater burden of respiratory severity markers (table S2).

In the comorbidity block, chronic cardiovascular disease and diabetes mellitus were substantially more frequent among Influenza A cases. Chronic cardiovascular disease was present in 23.23% of Influenza A cases (95% CI: 22.54–23.93), compared with 9.33% of Influenza B cases (95% CI: 8.11–10.72), yielding the largest difference among comorbidities. Diabetes mellitus was also more prevalent among Influenza A cases, affecting 13.97% (95% CI: 13.40–14.55), compared with 5.74% (95% CI: 4.78–6.87) among Influenza B cases. Other chronic pneumopathy, chronic neurological disease, chronic kidney disease, obesity, immunodeficiency/immunosuppression, and chronic liver disease also showed slightly higher prevalence among Influenza A cases (table S2).

In the preventive and therapeutic intervention block, COVID-19 booster vaccination was more frequent among Influenza A cases, with a prevalence of 24.30% (95% CI: 23.60–25.02), compared with 16.11% (95% CI: 14.53–17.82) among Influenza B cases. Influenza vaccination in the last campaign was also more frequent among Influenza A cases, whereas oseltamivir use was similar between groups, with prevalences of 21.61% (95% CI: 20.94–22.30) for Influenza A and 21.90% (95% CI: 20.10–23.80) for Influenza B (table S2).

In the clinical outcomes block, ICU admission was nearly identical between Influenza A and Influenza B cases, with prevalences of 28.03% (95% CI: 27.30–28.78) and 28.00% (95% CI: 26.03–30.05), respectively. However, mortality was higher among Influenza A cases. Death occurred in 12.74% of Influenza A cases (95% CI: 12.20–13.30), compared with 7.35% of Influenza B cases (95% CI: 6.27–8.61), corresponding to an absolute difference of 5.39 percentage points. This finding supports the interpretation that Influenza A was associated with a more severe hospitalized profile in 2024, characterized by a higher burden of chronic comorbidities, greater respiratory severity, and higher mortality (table S2).

**Table S2 –** Prevalence of demographic characteristics, symptoms, comorbidities, preventive and therapeutic interventions, and outcomes among hospitalized patients with Influenza A and Influenza B in Brazil, 2024.

| Variable | Influenza A  n (%) | 95% CI | Influenza B  n (%) | 95% CI | Difference A–B, pp |
| --- | --- | --- | --- | --- | --- |
| Demographic characteristic |  |  |  |  |  |
| Male sex | 6,825 (48.48) | 47.66–49.31 | 934 (48.70) | 46.46–50.93 | -0.21 |
| Symptoms and respiratory severity markers |  |  |  |  |  |
| Cough | 11,399 (80.98) | 80.32–81.62 | 1,530 (79.77) | 77.91–81.51 | 1.21 |
| Fever | 10,087 (71.66) | 70.91–72.39 | 1,475 (76.90) | 74.96–78.73 | -5.25 |
| Dyspnea | 8,824 (62.68) | 61.88–63.48 | 947 (49.37) | 47.14–51.61 | 13.31 |
| Respiratory distress | 8,441 (59.96) | 59.15–60.77 | 1,019 (53.13) | 50.89–55.35 | 6.83 |
| O2 saturation <95% | 7,398 (52.55) | 51.73–53.38 | 809 (42.18) | 39.99–44.40 | 10.37 |
| Fatigue | 2,731 (19.40) | 18.76–20.06 | 354 (18.46) | 16.78–20.26 | 0.94 |
| Sore throat | 2,045 (14.53) | 13.95–15.12 | 328 (17.10) | 15.48–18.85 | -2.57 |
| Vomiting | 1,835 (13.04) | 12.49–13.60 | 324 (16.89) | 15.28–18.64 | -3.86 |
| Diarrhea | 1,332 (9.46) | 8.99–9.96 | 194 (10.11) | 8.84–11.54 | -0.65 |
| Abdominal pain | 1,013 (7.20) | 6.78–7.63 | 184 (9.59) | 8.35–10.99 | -2.40 |
| Loss of smell | 236 (1.68) | 1.48–1.90 | 24 (1.25) | 0.84–1.86 | 0.43 |
| Loss of taste | 223 (1.58) | 1.39–1.80 | 28 (1.46) | 1.01–2.10 | 0.12 |
| Comorbidities |  |  |  |  |  |
| Chronic cardiovascular disease | 3,270 (23.23) | 22.54–23.93 | 179 (9.33) | 8.11–10.72 | 13.90 |
| Diabetes mellitus | 1,966 (13.97) | 13.40–14.55 | 110 (5.74) | 4.78–6.87 | 8.23 |
| Other chronic pneumopathy | 1,126 (8.00) | 7.56–8.46 | 65 (3.39) | 2.67–4.30 | 4.61 |
| Asthma | 1,059 (7.52) | 7.10–7.97 | 129 (6.73) | 5.69–7.94 | 0.80 |
| Chronic neurological disease | 754 (5.36) | 5.00–5.74 | 81 (4.22) | 3.41–5.22 | 1.13 |
| Immunodeficiency/immunosuppression | 526 (3.74) | 3.44–4.06 | 70 (3.65) | 2.90–4.59 | 0.09 |
| Chronic kidney disease | 502 (3.57) | 3.27–3.89 | 49 (2.55) | 1.94–3.36 | 1.01 |
| Obesity | 480 (3.41) | 3.12–3.72 | 36 (1.88) | 1.36–2.59 | 1.53 |
| Chronic hematologic disease | 187 (1.33) | 1.15–1.53 | 25 (1.30) | 0.88–1.92 | 0.02 |
| Down syndrome | 122 (0.87) | 0.73–1.03 | 21 (1.09) | 0.72–1.67 | -0.23 |
| Chronic liver disease | 105 (0.75) | 0.62–0.90 | 11 (0.57) | 0.32–1.02 | 0.17 |
| Preventive and therapeutic interventions |  |  |  |  |  |
| COVID-19 booster vaccination | 3,421 (24.30) | 23.60–25.02 | 309 (16.11) | 14.53–17.82 | 8.19 |
| Influenza vaccination | 1,990 (14.14) | 13.57–14.72 | 208 (10.84) | 9.53–12.32 | 3.29 |
| Oseltamivir use | 3,042 (21.61) | 20.94–22.30 | 420 (21.90) | 20.10–23.80 | -0.29 |
| Clinical outcomes |  |  |  |  |  |
| ICU admission | 3,946 (28.03) | 27.30–28.78 | 537 (28.00) | 26.03–30.05 | 0.03 |
| Death | 1,793 (12.74) | 12.20–13.30 | 141 (7.35) | 6.27–8.61 | 5.39 |

**Legend:** Values are presented as absolute numbers and percentages within each influenza type. The difference column represents the absolute percentage-point difference between Influenza A and Influenza B prevalence estimates. Positive values indicate higher prevalence among Influenza A cases, whereas negative values indicate higher prevalence among Influenza B cases. Ninety-five percent confidence intervals for prevalence estimates were calculated using the Wilson binomial method. Source: SIVEP-Gripe.

**Figure S1** – Comparative prevalence of clinical characteristics, comorbidities, preventive and therapeutic interventions, and outcomes among hospitalized patients with Influenza A and Influenza B in Brazil, 2024.

Legend: Bars represent the percentage prevalence of each variable within Influenza A and Influenza B cases. Variables with higher prevalence among Influenza A cases included dyspnea, low oxygen saturation, respiratory distress, chronic cardiovascular disease, diabetes mellitus, COVID-19 booster vaccination, influenza vaccination, and death. Variables with higher prevalence among Influenza B cases included fever, vomiting, sore throat, diarrhea, and abdominal pain. Source: SIVEP-Gripe.

After IPTW adjustment, covariate balance improved across most variables included in the propensity score model. Most post-weighting absolute standardized mean differences were below the conventional threshold of 0.10, indicating substantial improvement in balance between patients with and without COVID-19 booster vaccination. Residual imbalance remained for age, fever, chronic cardiovascular disease, and diabetes mellitus, which were therefore retained in the final weighted outcome model to reduce residual confounding. Detailed balance diagnostics are presented in Supplementary Table S3.

**Table S3 –** Covariate balance before and after inverse probability of treatment weighting for COVID-19 booster vaccination.

| Variable | Absolute SMD before IPTW | Absolute SMD after IPTW |
| --- | --- | --- |
| Age | 1.252 | 0.390 |
| Male sex | 0.181 | 0.080 |
| Fever | 0.327 | 0.130 |
| Cough | 0.025 | 0.025 |
| Sore throat | 0.028 | 0.055 |
| Dyspnea | 0.188 | 0.083 |
| Respiratory distress | 0.000 | 0.020 |
| Low oxygen saturation | 0.202 | 0.060 |
| Diarrhea | 0.108 | 0.034 |
| Vomiting | 0.221 | 0.062 |
| Chronic cardiovascular disease | 0.484 | 0.139 |
| Liver disease | 0.000 | 0.000 |
| Asthma | 0.038 | 0.038 |
| Diabetes mellitus | 0.356 | 0.112 |
| Chronic neurological disease | 0.084 | 0.044 |
| Immunodeficiency/immunosuppression | 0.102 | 0.000 |
| Obesity | 0.102 | 0.054 |
| Influenza vaccination | 0.244 | 0.028 |
| Oseltamivir use | 0.072 | 0.072 |

**Legend:** Absolute standardized mean differences were calculated before and after IPTW to evaluate covariate balance between patients with and without COVID-19 booster vaccination. Values below 0.10 after weighting were considered indicative of adequate balance. IPTW substantially improved covariate balance for most variables, although residual imbalance remained for age, fever, chronic cardiovascular disease, and diabetes mellitus. IPTW: inverse probability of treatment weighting; SMD: standardized mean difference.

The complementary calibration assessment showed a progressive increase in both predicted and observed mortality across quintiles of predicted risk. Mean predicted mortality increased from 1.56% in the lowest-risk quintile to 35.20% in the highest-risk quintile. Observed mortality followed a similar gradient, increasing from 1.00% to 34.00% across the same quintiles. Differences between observed and predicted mortality were small across strata, ranging from -1.20 to 2.44 percentage points, supporting acceptable calibration of the IPTW-weighted mortality model (table S4).

**Table S4 –** Calibration assessment of the IPTW-weighted mortality model across quintiles of predicted risk.

| Predicted-risk quintile | Weighted N | Mean predicted mortality, % | Observed mortality, % | Difference, percentage points |
| --- | --- | --- | --- | --- |
| Quintile 1, lowest risk | 5,785 | 1.56 | 1.00 | -0.56 |
| Quintile 2 | 5,788 | 4.08 | 3.00 | -1.08 |
| Quintile 3 | 5,787 | 8.54 | 10.00 | 1.46 |
| Quintile 4 | 5,787 | 17.56 | 20.00 | 2.44 |
| Quintile 5, highest risk | 5,786 | 35.20 | 34.00 | -1.20 |
| Total | **28,933** | **13.39** | **13.00** | **-0.39** |

**Legend:** Calibration was assessed by comparing mean predicted mortality with observed mortality across quintiles of predicted risk. Predicted mortality was derived from the IPTW-weighted logistic regression model. Observed mortality represents the weighted proportion of deaths within each predicted-risk quintile. Differences were calculated as observed mortality minus predicted mortality. This complementary calibration analysis was used alongside the Hosmer–Lemeshow goodness-of-fit test.
